# Effects of Macromolecular Crowding on Protein Conformational Changes

**DOI:** 10.1371/journal.pcbi.1000833

**Published:** 2010-07-01

**Authors:** Hao Dong, Sanbo Qin, Huan-Xiang Zhou

**Affiliations:** Department of Physics and Institute of Molecular Biophysics, Florida State University, Tallahassee, Florida, United States of America; University of California San Francisco, United States of America

## Abstract

Many protein functions can be directly linked to conformational changes. Inside cells, the equilibria and transition rates between different conformations may be affected by macromolecular crowding. We have recently developed a new approach for modeling crowding effects, which enables an atomistic representation of “test” proteins. Here this approach is applied to study how crowding affects the equilibria and transition rates between open and closed conformations of seven proteins: yeast protein disulfide isomerase (yPDI), adenylate kinase (AdK), orotidine phosphate decarboxylase (ODCase), Trp repressor (TrpR), hemoglobin, DNA β-glucosyltransferase, and Ap_4_A hydrolase. For each protein, molecular dynamics simulations of the open and closed states are separately run. Representative open and closed conformations are then used to calculate the crowding-induced changes in chemical potential for the two states. The difference in chemical-potential change between the two states finally predicts the effects of crowding on the population ratio of the two states. Crowding is found to reduce the open population to various extents. In the presence of crowders with a 15 Å radius and occupying 35% of volume, the open-to-closed population ratios of yPDI, AdK, ODCase and TrpR are reduced by 79%, 78%, 62% and 55%, respectively. The reductions for the remaining three proteins are 20–44%. As expected, the four proteins experiencing the stronger crowding effects are those with larger conformational changes between open and closed states (e.g., as measured by the change in radius of gyration). Larger proteins also tend to experience stronger crowding effects than smaller ones [e.g., comparing yPDI (480 residues) and TrpR (98 residues)]. The potentials of mean force along the open-closed reaction coordinate of apo and ligand-bound ODCase are altered by crowding, suggesting that transition rates are also affected. These quantitative results and qualitative trends will serve as valuable guides for expected crowding effects on protein conformation changes inside cells.

## Author Summary

The biophysical properties of proteins inside cells can be expected to be quite different from those typically measured by *in vitro* experiments in dilute solutions. In particular, intracellular macromolecular crowding may significantly affect the equilibria and transition rates between different conformations of a protein, and hence its functions. What are the trends and magnitudes of such crowding effects? We address this question here by applying a recently developed approach for modeling crowding. Seven proteins, each with structures for both an open state and a closed state, are studied. Crowding exerts significant effects on the open-closed equilibria of four proteins and more modest effects on the remaining three. Potentials of mean force along the open-closed reaction coordinate, and hence transition rates, are similarly affected. The extent of conformational changes is the main determinant for the magnitudes of crowding effects, but the protein size also plays an important role. The effects of crowding become stronger as the protein size increases. Conformational transitions of the ribosome, an extremely large complex, during translation are predicted to experience particularly strong effects of intracellular crowding. We conclude that deduction of intracellular behaviors from *in vitro* experiments requires explicit consideration of crowding effects.

## Introduction

**Figure 1:**
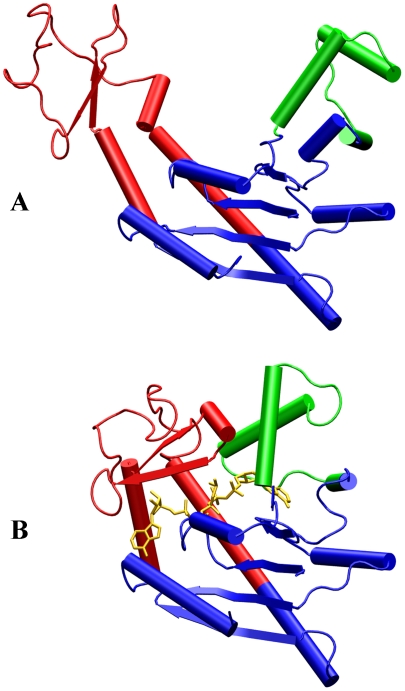
Structures of AdK in the open and closed states. (A) The substrate-free open state (PDB code 4AKE). (B) The closed state (PDB code 1AKE) in the presence of inhibitor Ap_5_A. The ATP-binding domain is in red, the AMP-binding domain is in green, the inhibitor is in yellow, and the CORE domain is in blue.

It is increasingly recognized that protein dynamics serves the critical link between structure and function [1]–[6]. An important manifestation of protein dynamics is the sampling of alternative conformations. These conformational changes can be triggered by substrate (or ligand) binding [7] and post-translational modifications such as phosphorylation [8]. Increasingly, structures of the same proteins at different functional states are becoming available. These structures provide atomistic details of conformational changes. For example, adenylate kinase (AdK), an enzyme that catalyzes the phosphoryl transfer from ATP to AMP, undergoes a significant conformational transition, from an “open” conformation in the apo form and to a “closed” conformation in the ligand-bound form [9]–[11]. The main differences between these two conformations occur in the ATP- and AMP-binding domains, with the CORE domain relatively rigid (Figure 1). Other examples with well-characterized conformational transitions include proteins responsible for signal transduction across cell membranes [12]–[14] and ion channels [15].

Biophysical characterizations of protein conformational changes have mostly been carried out under dilute conditions. However, the environments where proteins perform their biological functions, i.e., extracellular space, cell membrane, and cytoplasm, are crowded with macromolecules. For example, the cytoplasm of *Escherichia coli* contains about 300–400 g/l of macromolecules [16], which are estimated to occupy over 30% of the total volume. In cell membranes, membrane proteins occupy a similar level of the total surface area [17]. How the crowded cellular environments affect the equilibria and transition rates between different conformations of proteins is still poorly understood. Qualitatively, one expects that macromolecular crowding will significantly modify the energy landscapes of conformational changes, favoring more compact structures over more open ones [18]. Such effects of crowding have been scrutinized experimentally (see [18] for a recent review). Molecular dynamics simulations have also been carried out to investigate the energy landscapes of a number of proteins under crowding, in the context of either conformational change [19] or folding-unfolding transition [20]–[22]. To speed up conformational sampling, the proteins in these studies were represented at a coarse-grained level.

Recently we have developed an approach [23], [24], referred to as postprocessing, which opens the door to atomistic modeling of proteins under crowding. In this approach, the motions of a test protein are simulated in the absence of crowders. Conformations from this simulation are then used to calculate the change in chemical potential if they are transferred to a crowded solution. The dependence of the change in chemical potential on reaction coordinates then captures the influence of crowding on the energy landscape. The postprocessing approach has been applied to study effects of crowding on protein folding and binding stability [23], [25], [26] and on the open-closed equilibrium of the HIV-1 protease dimer [24]. In the latter application it has been shown that the postprocessing approach yields results identical to those obtained from direct simulations of the protein in the presence of crowders [19], [24].

Here we apply the postprocessing approach to investigate the impact of macromolecular crowding on the open-closed equilibria of seven proteins: AdK [9], [10], yeast protein disulfide isomerase (yPDI) [27], [28], orotidine phosphate decarboxylase (ODCase; functioning as a dimer) [29], Trp repressor (TrpR) [30], hemoglobin (Hb) [31], DNA β-glucosyltransferase (BGT) [32], and Ap_4_A hydrolase (Ap_4_Aase) [33], [34]. The biological functions and subcellular locations of these proteins are listed in Table 1. We find crowding to reduce the open-to-closed population ratios to various extents; the potentials of mean force (PMFs) along the open-closed reaction coordinate of apo and ligand-bound ODCase, and hence the transition rates, are similarly affected. The biological implications of these results are discussed below.

**Table 1:** Biological functions and subcellular locations of seven proteins.

Protein	Organism	Subcellular location	Biological Function	PDB code
AdK	*Escherichia coli*	cytoplasm	ATP-AMP transphosphorylase	4AKE & 1AKE
yPDI	*Saccharomyces cerevisiae*	lumen of endoplasmic reticulum	catalyses rearrangement of protein disulfide bonds	3BOA & 2B5E
ODCase	*Saccharomyces cerevisiae*	cytoplasm	converts orotidine mono- phosphate to uridine mono-phosphate, the last step in biosynthesis of pyrimidine nucleotides	1DQW & 1DQX
TrpR	*Escherichia coli*	cytoplasm	regulates transcription in tryptophan biosynthesis	1WRP & 3WRP
Hb	*Homo sapiens*	cytoplasm	oxygen transport	2DN1 & 2DN2
BGT	*Enterobacteria phage T4*	cytoplasm of host cell	catalyzes transfer of glucose from uridine diphosphoglucose to hydroxymethylcytosine in DNA	1JEJ & 1JG6
Ap_4_Aase	*Lupinus angustifolius*	cytoplasm	catalyses cleavage of diadenosine tetraphosphate into ATP and AMP	1F3Y & 1JKN

## Results/Discussion

The X-ray structures of AdK in the open and closed states are shown in Figure 1; comparison of open and closed structures for all the seven proteins is found in Figure S1. For each protein, molecular dynamics simulations of the open and closed states are separately run. Representative open and closed conformations are then used to calculate the crowding-induced changes in chemical potential for the two states. The difference in chemical-potential change between the two states finally predicts the effects of crowding on the population ratio of the two states.

We study a range of crowding conditions, as defined the radius, *R*_c_, and the volume fraction, *φ*, of the crowders. The value of *R*_c_ ranges from 15 to 50 Å and the value of *φ* ranges from 5 to 35%. These ranges of crowder sizes and volume occupancies likely cover the conditions of cytoplasmic milieus [35]. Among the seven proteins studied, four are found to experience significant crowding effects on the open-to-closed population ratios while the remaining three experiencing more modest effects.

### Proteins experiencing significant crowding effects

**Figure 2:**
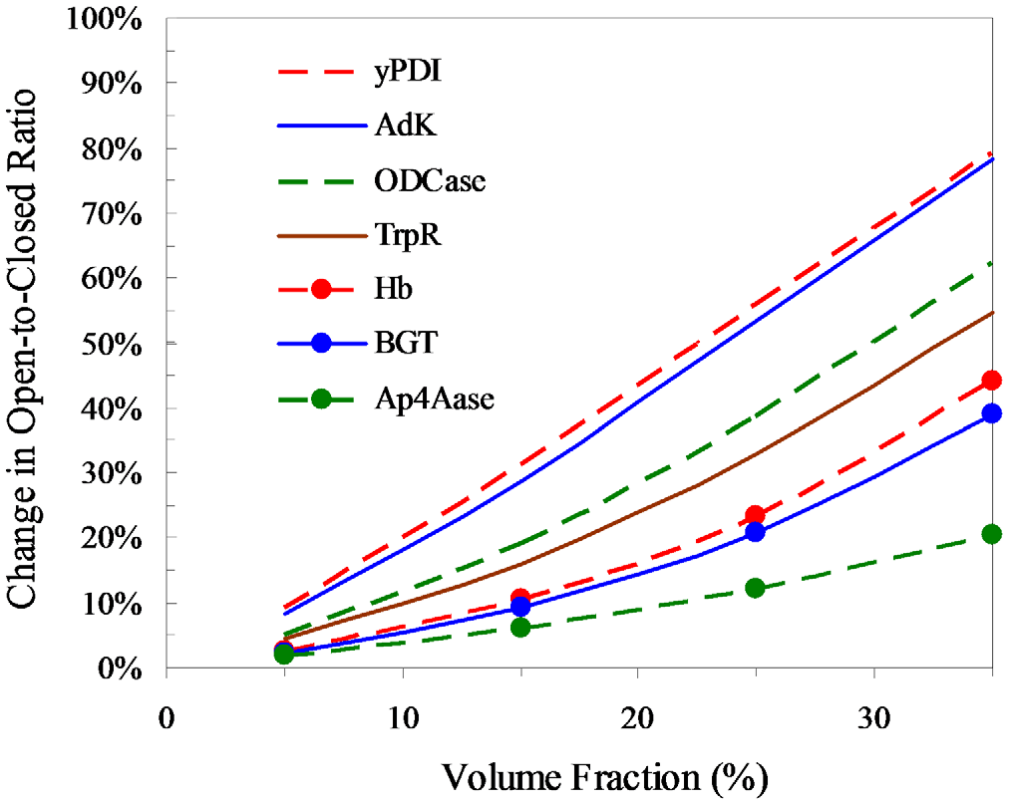
Effects of crowding on the open-to-closed population ratios of seven proteins. The crowder radius is 15 Å.

Relatively strong effects of crowding are found on the open-to-closed population ratios of four proteins: AdK, yPDI, ODCase, and TrpR. In Figure 2 we show the relative changes in the open-to-closed population ratios of the four proteins by 15-Å crowders at various volume fractions. Corresponding results for 30-Å crowders are shown in Figure S2.

We describe the calculation results on AdK in some details. Previous computational and experimental studies have determined the pathways and rates of the open-closed transitions for this protein [36]–[39]. In dilute solutions, the open-to-closed population ratio is ∼3 in the ligand-free form and ∼1/5 in the ligand-bound form [36], [39]. In the presence of 15-Å crowders occupying 35% of volume, the open-to-closed population ratio is reduced by 4.6-fold, or, equivalently, 78%, making the open state less stable than the closed one even in the ligand-free form. The reduction in the open-to-closed population ratio comes about because it is harder to accommodate the open conformations than the closed conformations in the crowded solution. The probabilities of successful placement for the open and closed states are 2.96×10^−8^ and 1.36×10^−7^, respectively (see Figure S3).

At a given volume fraction, the effects of crowding are larger for smaller crowders than for larger crowders. For example, at 35% volume fraction, crowding reduces the open-to-closed population ratio of AdK by 78%, 59%, 35%, and 17%, respectively, when the crowder radii are 15, 20, 30, and 50 Å. Comparison of the results for 15-Å crowders in Figure 2 and the counterparts for 30-Å crowders in Figure S2 shows the same trend. This dependence on crowder size has been seen previously in studies of protein folding, binding, and conformational change [23]–[26]. As we noted [23], only a small number of large crowders are needed to occupy the same volume as a large number of small crowders. Even though the “obstacles” amount to the same total volume, the former arrangement is more compact than the latter, and hence easier to accommodate a test protein. In other words, the latter arrangement is more discriminating between the open conformations and closed conformations of a protein, and therefore produces a stronger effect on the open-to-closed population ratio.

Qualitatively similar effects of crowding are found for the other three proteins. In the presence of 15-Å crowders occupying 35% of volume, the open-to-closed population ratios of yPDI, ODCase and TrpR are reduced by 79%, 62% and 55%, respectively. These are to be compared with the 78% reduction reported above. Clearly, the open-closed transitions of all these four proteins are prone to crowding effects expected of their intracellular environments.

### Proteins experiencing modest crowding effects

As shown in Figure 2, the effects of crowding on the open-to-closed ratios of the other three proteins, Hb, BGT, and Ap_4_Aase, are modest. In the presence of 15-Å crowders occupying 35% of volume, the open-to-closed population ratios of these proteins are reduced by 44%, 39%, and 20%, respectively.

It should be noted that the modest effects on the open-to-closed population ratios come about not because crowding does not exert significant effects on the open or closed state, but rather because the effects exerted on the open and closed states are similar. For example, at *R*_c_ = 15 Å and *φ* = 35%, the probabilities of successful placements for the open and closed states of Hb are 3.26×10^−15^ and 5.83×10^−15^, respectively. These values are seven orders of magnitude smaller than the corresponding quantities for AdK reported above (due to the much larger size of Hb). However, here the values for the open and closed states are very similar, leading to a modest effect on the open-to-closed population ratio.

### Determinants for the magnitudes of crowding effects

What explains the large variations in crowding effects reported above? For a given protein, what matters is the difference in the effects exerted on the open state and the closed state. Therefore we expect the extent of conformational changes between the two states to be a main determinant. We use two quantities to measure the conformational changes: the solvent accessible surface area (SASA) and the radius of gyration, *R*_g_. In Table 2 we list the relative differences in SASA and in *R*_g_ for the seven proteins. It is clear that the four proteins that experience significant crowding effects (AdK, yPDI, ODCase, and TrpR) have large differences in SASA and *R*_g_ between the open and closed states. On the other hand, relatively small differences in SASA and *R*_g_ are found for the remaining three proteins (Hb, BGT, and Ap_4_Aase), which experiences more modest crowding effects. In one extreme, in the case of AdK, both measures differ by over 10% between the open and the closed states, whereas in the other extreme, in the case of Hb, both measures differ by less than 2%. We may thus conclude that the extent of conformational changes is the main determinant for the magnitudes of crowding effects.

**Table 2:** Number of residues (*N*_res_) for each of seven proteins and solvent accessible surface area (SASA) and radius of gyration (*R*_g_) in either open or closed state.

	*N*_res_	SASA (Å^2^)			*R*_g_ (Å)		
		Open state	Closed State	Rel. Diff. (%)	Open state	Closed State	Rel. Diff. (%)
AdK	214	12441	11179	11.3	19.1	16.7	14.1
yPDI	480	29080	27484	5.8	23.8	21.7	9.6
ODCase	534	22972	21544	6.0	24.6	23.8	3.4
TrpR	98	7795	7211	8.1	14.9	14.0	6.6
Hb	574	24631	24522	0.4	23.7	23.3	1.7
BGT	351	17609	16918	4.1	22.5	21.8	2.9
Ap_4_Aase	165	9743	9712	0.3	16.4	15.8	3.5

*N*_res_ is the total number of residues identical in the open and closed structures. For each protein except ODCase in either the open or closed state, averages of SASA and *R*_g_ calculated over 100 representative conformations are shown; variance for each entry is <1.5% of the average. For ODCase, further averaging over four independent trajectories is taken.

We have emphasized the fact that, for a given protein, the effect of crowding depends on the size of the crowders (when the volume fraction is kept constant). Conversely, for the same crowder size and crowder volume fraction, it can be expected that larger proteins will experience stronger crowding effects. This expectation is borne out by our calculation results. In particular, yPDI and TrpR show similar differences in SASA and *R*_g_ between their open and closed states but differ in size. yPDI has ∼5 times more residues than TrpR. Correspondingly the crowding effects experienced by yPDI is ∼2-fold higher than those by TrpR (Figure 2). Similarly the difference in crowding effects between Hb and Ap_4_Aase can be partly attributed to their size difference (574 vs. 165 residues).

### Crowding effects on conformational transition rates

**Figure 3:**
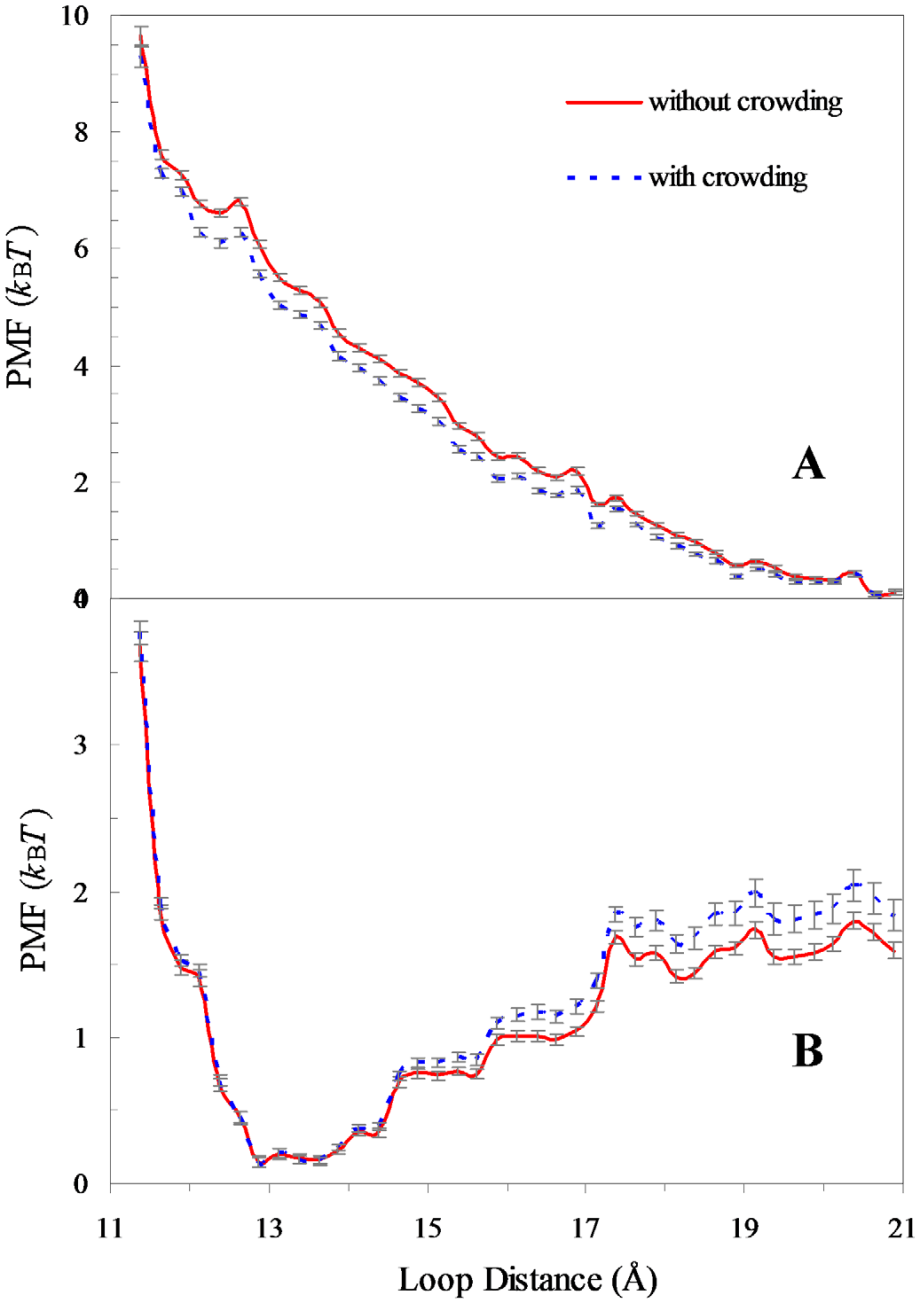
Potentials of mean force along the open-closed reaction coordinate of ODCase in the absence and presence of crowding. (A) The apo form. (B) The BMP-bound form.

Crowding not only affects the equilibria of open and closed states but is also expected to affect their transition rates. We study the latter problem on ODCase. The open and closed structures of this protein were solved in the absence and presence of a transition-state analogue, 6-hydroxyuridine 5′-phosphate (BMP), respectively [29]. The two structures differ significantly in the distance between two loops around the active site (Figure S1C), which is ∼20 and 12.5 Å, respectively. The PMFs of the apo and BMP-bound forms along the loop distance are calculated in the absence of crowding and shown in Figure 3. In the apo form, the transition from the open state (at loop distance ∼20.5 Å) to the closed state (at loop distance ∼12.5 Å) encounters an energy barrier ∼6.7 *k*_B_ *T*, where *k*_B_ is Boltzmann's constant and *T* is the absolute temperature. In the BMP-bound form, the transition from the closed state to the open state encounters a series of barriers, the most significant of which is at ∼1.5 *k*_B_ *T*.

Using the postprocessing approach we calculate the PMFs in the presence of crowding. The results for the crowding condition of *R*_c_ = 15 Å and *φ* = 35% are also shown in Figure 3. It can be seen that the energy barrier for the open-to-closed transition in the apo form is lowered, by ∼0.5 *k*_B_ *T*, which corresponds to a 65% increase in transition rate. On the other hand, the most significant energy barrier for the closed-to-open transition is raised, by ∼0.2 *k*_B_ *T*, corresponding to a 22% decrease in transition rate. These effects of crowding on the energy barriers, while relatively moderate, are nevertheless statistically significant (see error bars in Figure 3).

### Biological implications

Proteins dynamics provides the critical link between structure and function. In present study, we have investigated how the crowded environments inside cells might affect protein dynamics. Our results have important implications for the functions of the proteins studied here and for *in vivo* biological functions in general.

In catalyzing the phosphoryl transfer from ATP to AMP, AdK undergoes large conformational changes. In dilute solutions, the open conformation is favored in the apo form whereas the closed conformation is favored when the substrates are bound. However, open-closed transitions occur in both the apo form and the substrate-bound form [36], [39]. Moreover, the closed-to-open transition in the substrate-bound form is thought to limit the overall catalytic rate (under substrate saturation) [36]. Our calculations show that crowding will significantly shift the open-closed equilibrium toward the closed state, such that the closed state may become favored even in the apo form. Qualitatively it can be expected that, under crowding, the rate of the closed-to-open transition in the substrate-bound form, i.e., the putative rate-limiting step of the catalytic reaction, will be reduced. Intracellular crowding will thus have a significant impact on the catalysis activity of AdK. Future work on the transition state of the open-closed transitions will allow us to quantitatively assess the effects of crowding on the transition rates.

The case of ODCase is similar. Conformational changes, especially closure and opening of the loops around the active site, are essential for substrate binding and product dissociation. A recent kinetic study by Wood et al. [40] indicates that loop closure and opening are partially rate-limiting for *k*_cat_/*K*_M_ and *k*_cat_, respectively, in dilute solutions. Our calculations show that crowding stabilizes the closed state. Furthermore, calculations of the PMFs along the loop distance show that crowding increases the rate of loop closure in the apo form and hence *k*_cat_/*K*_M_ but decreases the rate of loop opening in the ligand-bound form and hence *k*_cat_. Interestingly, these predicted trends were actually observed by Wood et al. when the kinetic study was done in the presence of Ficoll, a common crowding agent.

The considerable conformational flexibility of yPDI, which is essential for the formation of the correct pattern of disulfide bonds in substrate proteins, has been demonstrated by two structures determined from crystals grown at two different temperatures, 4°C and 22°C [27], [28]. The protein consists of four domains, labeled as a, b, b′, and a′ (see Figure S1B). Both the a and a′ domains harbor an active site. The 4-°C structure is more compact, forming a twisted U shape with the b and b′ as the base and the a and a′ domains as the arms. The 22-°C structure is more open, with the a and a′ domains showing significant rearrangement relative to the b and b′ domains, resulting in a boat shape. The conformational flexibility may be important in affording yPDI the ability to accommodate substrate proteins with different sizes. It may also be important in promoting the activity of yPDI on each substrate. Indeed, with ether reduced or scrambled ribonuclease as substrate in dilute solutions, the activity of yPDI is reduced when the arrangement of the a and a′ domains relative to the b and b′ domains is locked in that of the twisted U shape by new disulfide bonds [28]. Moreover, the same restriction on domain rearrangement also reduces the activity of yPDI *in vivo*. Our calculations show that crowding favors the compact U conformation over the open boat conformation, thereby reducing the conformational flexibility. In addition, crowding promotes protein oligomerization [23], [25]. yPDI forms a weaker dimer in dilute solutions [28], but the propensity to dimerize may be significantly enhanced in the crowded environment of its cellular location, the lumen of the endoplasmic reticulum. (In a model for the dimer as present in the 22°C crystal structure, the a′ domain active site becomes buried whereas the a domain active site is still solvent exposed [28].) Dimer formation has indeed been detected in the endoplasmic reticulum [28]. yPDI exhibits a difference between *in vitro* and *in vivo* activities: the a′ domain active site is more potent than the a domain active site for a native substrate *in vitro*, but the reserve is true *in vivo* [41]. *In vivo* dimer formation suggests a plausible explanation for this difference. Intrinsically the a′ domain active site may be more potent, but in the endoplasmic reticulum yPDI may most exist as dimer, in which the a′ domain active site is buried and hence inaccessible to the substrate whereas the a domain active site is accessible to the substrate.

The transition between the R state and the T state is essential for Hb to carry out its function of transporting oxygen from the lungs to the tissues. The R state is slightly more “open” than the T state and has high oxygen affinity; the T state has low oxygen affinity. In red blood cells, Hb has concentrations ∼300 g/l, amounting to ∼35% of the cell volume. Modeling Hb molecules as spheres with a radius ∼30 Å, our calculations show that intracellular crowding leads to a modest 10% increase in the T-to-R population ratio and correspondingly a small decrease in the oxygen affinity of Hb. Given that the oxygen affinity of Hb is strongly regulated by pH, CO_2_, Cl^−^, and D-2,3-bisphosphoglycerate [42], the small decrease in oxygen affinity expected of intracellular crowding is probably not of physiological significance. We note in passing that a variant of normal Hb, sickle Hb, can polymerize when deoxygenated, leading to deformation of red blood cells, vascular occlusion, and anemia. It has been demonstrated that macromolecular crowding has a dramatic effect on sickle Hb polymerization [43].

Our calculations show that the extent of conformational changes is the main determinant for the magnitudes of crowding effects. The study here has focused on structured proteins. We note that intrinsically disordered proteins should undergo even greater conformational changes, and are thus expected to experience even stronger effects of macromolecular crowding. Indeed, the intrinsically disordered α-synuclein undergoes a temperature-induced collapsed-to-expanded transition in dilute solutions, but such a transition is prevented in a solution crowded by a bystander protein and in living *Escherichia coli* cells [44]. α-synuclein has a tendency to aggregate into fibrils, which is the underlying cause for Parkinson's disease; the delay time before fibrillation is considerably shortened by macromolecular crowding [45], [46].

Our calculations also show that protein size plays an important role in the magnitudes of crowding effects. Larger proteins are expected to experience stronger crowding effects. One of the largest systems in the Protein Data Bank (PDB) is that of the ribosome, which is the machinery for protein translation. During translation, the ribosome undergoes a series of conformational changes; the structures of the ribosome in many of these functional states have now been determined [47]. Our preliminary calculation indicates that the relative stability between these states may be significantly changed by intracellular crowding. The largest change exerted by crowding in the relative stability between the open and closed states of the seven proteins studied here is 1.6 *k*_B_ *T*. The same crowding condition favors the conformational change of the ribosome upon binding of a release factor by ∼5 *k*_B_ *T*. The particularly strong crowding effects predicted for the conformational transitions of the ribosome perhaps partly explain why protein translation in an *in vitro* setting [48] is not as efficient as *in vivo* [49].

In conclusion, the preceding discussion makes it clear that intracellular crowding will significantly affect conformational changes and biological functions of proteins and molecular machines. Consequently, deduction of intracellular behaviors from *in vitro* experiments requires explicit consideration of crowding effects.

## Methods

### Postprocessing approach for modeling crowding

We briefly summarize our approach for atomistic modeling of crowding effects [23]. The aim here is to calculate the crowding-induced change in the free-energy difference, ΔΔ*G*, between the open and closed states of a protein. If the change in chemical potential when the protein in the open state is transferred from a dilute solution to a crowded solution is Δ*μ*_o_ and the corresponding quantity in the closed state is Δ*μ*_c_, then
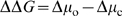
As the measure of the effect of crowding, we report the relative change, *κ*, in the open-to-closed population ratio by crowding, given by
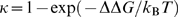



Here, like in previous molecular-dynamics studies of macromolecular crowding [19]–[22], we model the interactions between the test protein and crowders as hard-core repulsion. In that case, Δ*μ*, the change in chemical potential in a given state due to crowding, is related to the probability, *f*, that the protein in that state can be successfully placed into a box of randomly distributed crowders:
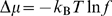
Following previous studies [19]–[22], we further model the crowders as spherical particles. We have developed an algorithm for calculating *f* [23], hereafter referred to as the particle-insertion algorithm, since it is similar in spirit to Widom's particle-insertion method [50].

In our previous study of crowding effects on the open-closed equilibrium of the HIV-1 protease dimer [24], the aim was to demonstrate that the postprocessing approach yields results identical to those obtained from direct simulations of the protein in the presence of crowders [19]. The open and closed conformations in the absence of crowding, used for postprocessing, were obtained from a single simulation; we were able to simulate transitions between the two states because the protein was represented at a coarse-grained level. In the present study, we simulate the open state and closed state separately and thus avoid the slow transitions between them. (The separate simulations of end states follow our previous studies of protein folding and binding [23], [25], [26].) Postprocessing the conformations from the two separate simulations allows us to determine the effects of crowding on the open-to-closed population ratio.

The particle-insertion algorithm has been used to implement the postprocessing approach in all our previous studies [23]–[26]. More recently we have discovered that Δ*μ* can be predicted theoretically [51], thus significantly speeding up the postprocessing approach. The gain in computational speed is especially important when the size of protein increases, whereupon the values of *f* become exceedingly small and hence difficult to obtain using the particle-insertion algorithm. In brief, the prediction of Δ*μ* is based on generalizing the fundamental measure theory [52], which is designed for convex test particles and crowders, to atomistic proteins interacting with spherical crowders. The theory predicts Δ*μ* as a linear function of the volume *v*_p_, surface area *s*_p_, and linear size *l*_p_ of the test protein:
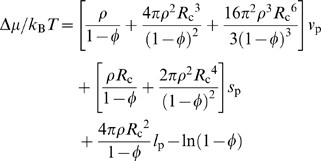
where *ρ* is the number density of the crowders. In the generalized fundamental measure theory (GFMT) [51], *v*_p_, *s*_p_, and *l*_p_ are defined according to the so-called crowder-exclusion surface. This surface is similar to Richard's molecular surface [53], but with the probe radius set to the radius of the crowders. The average values of *v*_p_, *s*_p_, and *l*_p_ calculated on the open and closed conformational ensembles of the seven proteins are listed in Table S1. We do not use these average values to predict the final Δ*μ* result. Instead, we use the particular *v*_p_, *s*_p_, and *l*_p_ for each conformation to calculate a Δ*μ* value and then Boltzmann-average the individual Δ*μ* values over the conformational ensemble to yield the final Δ*μ* result. In Figure S3, we show that the GFMT predictions for Δ*μ*_o_ and Δ*μ*_c_ of AdK agree very well with the results obtained by the particle-insertion algorithm. Note that, for the crowding condition of *R*_c_ = 15 Å and *φ* = 35%, convergent results could not be obtained by the particle-insertion algorithm. All the results reported in the main text are calculated by the GFMT.

### Molecular dynamics simulations

For each protein, molecular dynamics simulations of the open and closed states are separately run using the Amber program. In each simulation, the protein molecule starts from the X-ray or NMR structure (with PDB code given in Table 1) and is solvated in TIP3P water molecules. The simulations are run at constant temperature (300 K) and constant pressure (1 bar), with the particle mesh Ewald method used to treat long-range electrostatic interactions. The total time of each simulation is 10 ns; 100 conformations are evenly selected from the last 8 ns as representatives of the conformational ensemble. Root-mean-square-deviations (RMSDs; measured on C_α_ atoms) of these representative conformations from the starting X-ray or NMR structure are typically ∼1.5 Å, but the open states of AdK and yPDI and the closed state of TrpR show higher RMSDs (see Figure S4).

To investigate how representative the conformational ensemble from a single trajectory is of a protein in a given state, four independent trajectories each of ODCase in the open state and the closed state are run. Crowding effects calculated on the four independent ensembles are similar. For example, the probabilities of successful placement for ODCase in the closed state calculated on the four ensembles are (1.89±0.34)×10^−14^ under the crowding condition of *R*_c_ = 15 Å and *φ* = 35%.

For some proteins, there are small differences in the residues present in the starting structures of the open and closed states. Such differences would make an artificial contribution to ΔΔ*G*; we eliminate this artifact by keeping only the residues that are present in both the open and closed conformations in calculations of Δ*μ*.

### Solvent accessible surface area

Solvent accessible surface area is calculated by using the NACCESS program [54], with a 1.4-Å probe radius.

### Potentials of mean force

The PMFs along the open-closed reaction coordinate are calculated by umbrella sampling. The reaction coordinate, *x*, for ODCase is taken as the distance between the centers of mass of two loops, consisting of residues 151–161 and residues 203–218, respectively (Figure S1C). The umbrella sampling consists of 32 windows covering *x* values from 11.5 Å to 20.8 Å (with 0.3 Å increment). Harmonic restraints with force constants of 5 and 10 kcal/mol/Å^2^, respectively, are used for the apo form and the BMP-bound form, respectively. For the apo form, simulations in different restraint windows are independent; the simulation in each window consists of 0.2 ns of equilibration and 1.3 ns of production, with 1625 conformations used for PMF calculations. For the BMP-bound form, the simulations are carried out sequentially, with the conformation after 0.2 ns of equilibration in one window saved for the starting structure in the next window; 3500 subsequent conformations in the next 2.8 ns of simulation are used for PMF calculations.

The weighted histogram analysis method (WHAM) [55], [56] was used to obtain the PMFs. A PMF, *W*(*x*), is related to the probability density in *x*, *P*(*x*), via *P*(*x*) = exp[−*W*(*x*)/*k*_B_ *T*]/*C*, where *C* is a normalization constant. In the standard WHAM, the probability density at discrete *x* values, *x_j_*, is obtained by iterating the following two equations to convergence:
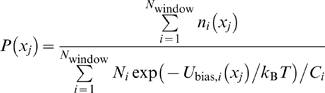


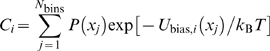
where *i* is the index for restraint windows; *N*_window_ is the total number of such windows; *U*_bias,*i*_(*x*) is the bias potential in window *i*; *N_i_* is the total number of sampled conformations from the window *i* simulation; *C_i_* is a normalization constant for window *i*; *N*_bin_ is the total number of *x* values for which *P*(*x*) is calculated; and *n_i_*(*x_j_*) is the number of conformations from the window *i* simulation which have *x* values belong to the *x_j_* bin. Errors are calculated by bootstrapping (http://membrane.urmc.rochester.edu).

From the conformations sampled in the absence of crowding, we use the postprocessing approach to obtain the PMF in the presence of crowding. Specifically, we assign each sampled conformation a statistical weight due to crowding; this statistical weight is *f*, the probability of successful placement into a box of crowders. Note that *N*_i_ in the above equation can be viewed as the sum over a statistical weight of 1 for each conformation from the window *i* simulation; similarly *n_i_*(*x_j_*) can be viewed as a sum over a statistical weight of 1 for each conformation from the window *i* simulation with an *x* value in bin *x_j_*. For the PMF in the presence of crowding, *N_i_* is replaced by the sum over the *f* values of the conformations from the window *i* simulation, and *n_i_*(*x_j_*) is replaced by the sum over the *f* values of the conformations from the window *i* simulation which have *x* values in bin *x_j_*.

ODCase functions as a homodimer but the two active sites are catalytically independent [57]. In our umbrella sampling, for each window the loop distances in the two subunits are restrained to the same value. For each sampled conformation, the actual loop distances of the two subunits were first averaged and then the result was used to find the corresponding bin *x_j_*. In addition, two independent sets of umbrella sampling simulations are carried out for both the apo form and the BMP-bound form; their averages are reported in Figure 3.

## Supporting Information

Figure S1 Structural differences between open and closed states of seven proteins. (A) AdK. (B) yPDI. (C) ODCase. (D) TrpR. (E) Hb. (F) BGT. (G) Ap_4_Aase. For each protein, the open structure is in blue and the closed structure is in red; the two structures are superimposed on regions that exhibit relatively small changes. The PDB codes for these structures are listed in Table 1.
(3.02 MB TIF)

Figure S2 Effects of crowding on the open-to-closed population ratios of seven proteins. The crowder radius is 30 Å.
(0.54 MB TIF)

Figure S3 Comparison of GFMT predictions and results obtained by the particle-insertion algorithm. (A) Crowding-induced change, Δμ_o_, in the chemical potential of AdK in the open state. (B) Corresponding quantity in the closed state. The crowder radius is 15 Å.
(0.52 MB TIF)

Figure S4 Root-mean-square-deviations of conformations during simulations from the starting X-ray or NMR structures. (A) AdK. (B) yPDI. (C) ODCase. (D) TrpR. (E) Hb. (F) BGT. (G) Ap_4_Aase. For each protein, C_α_ RMSDs of the open and closed states are displayed in blue and red, respectively. For ODCase, results averaged over four independent trajectories are shown.
(0.55 MB TIF)

Table S1 Geometrical parameters of seven proteins for the prediction of Δμ_o_ and Δμ_c_ by the generalized fundamental measure theory
(0.07 MB DOC)
