# Supplementary figures and images for: Effects of Macromolecular Crowding on Protein Conformational Changes
Source: PLoS Comput Biol. 2010 Jul 1;6(7):e1000833. doi: 10.1371/journal.pcbi.1000833 (PMC2895631; doi:10.1371/journal.pcbi.1000833)

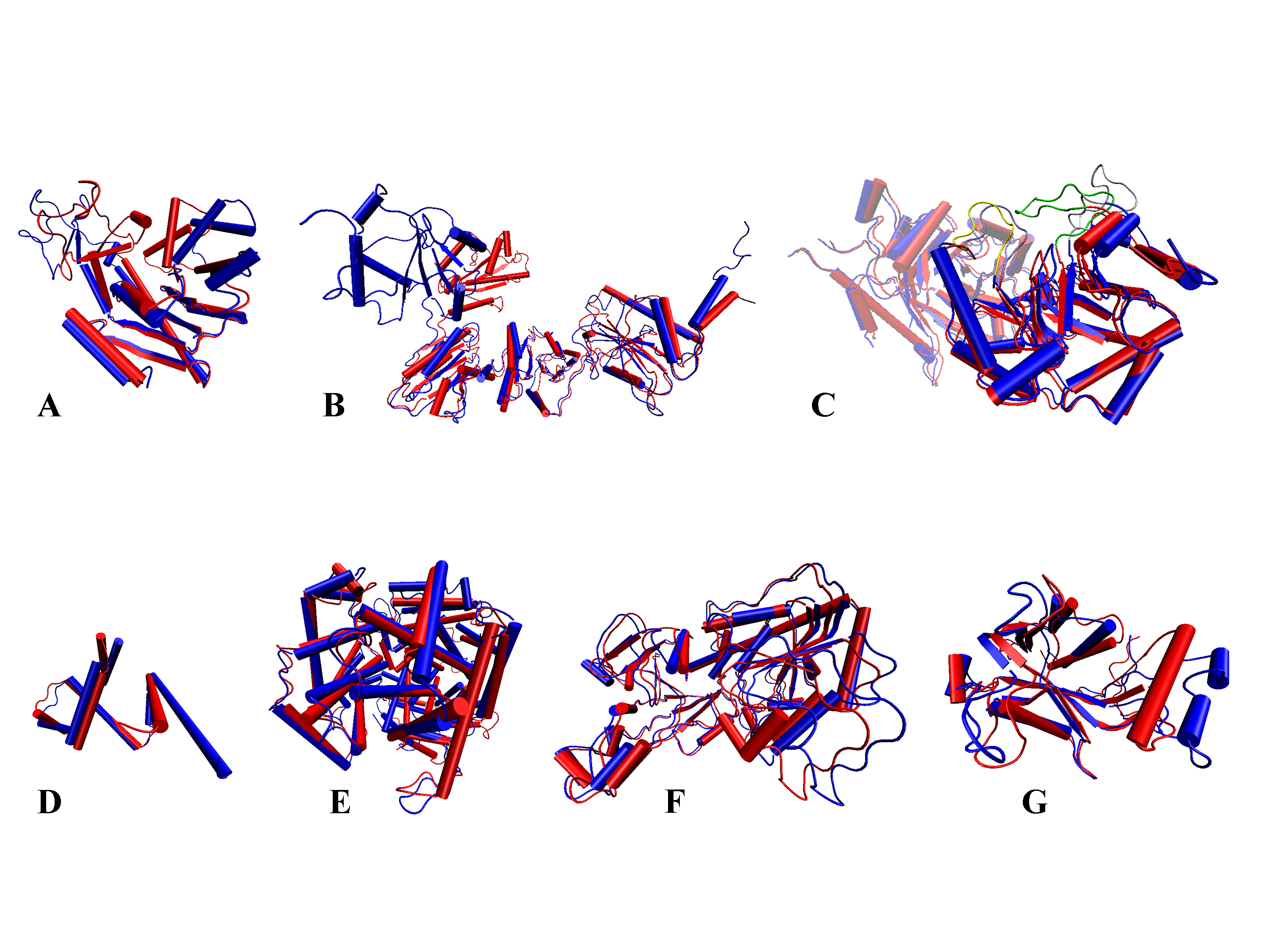

Supplement: Figure S1 — Structural differences between open and closed states of seven proteins. (A) AdK. (B) yPDI. (C) ODCase. (D) TrpR. (E) Hb. (F) BGT. (G) Ap4Aase. For each protein, the open structure is in blue and the closed structure is in red; the two structures are superimposed on regions that exhibit relatively small changes. The PDB codes for these structures are listed in Table 1. (3.02 MB TIF) [file pcbi.1000833.s001.tif]

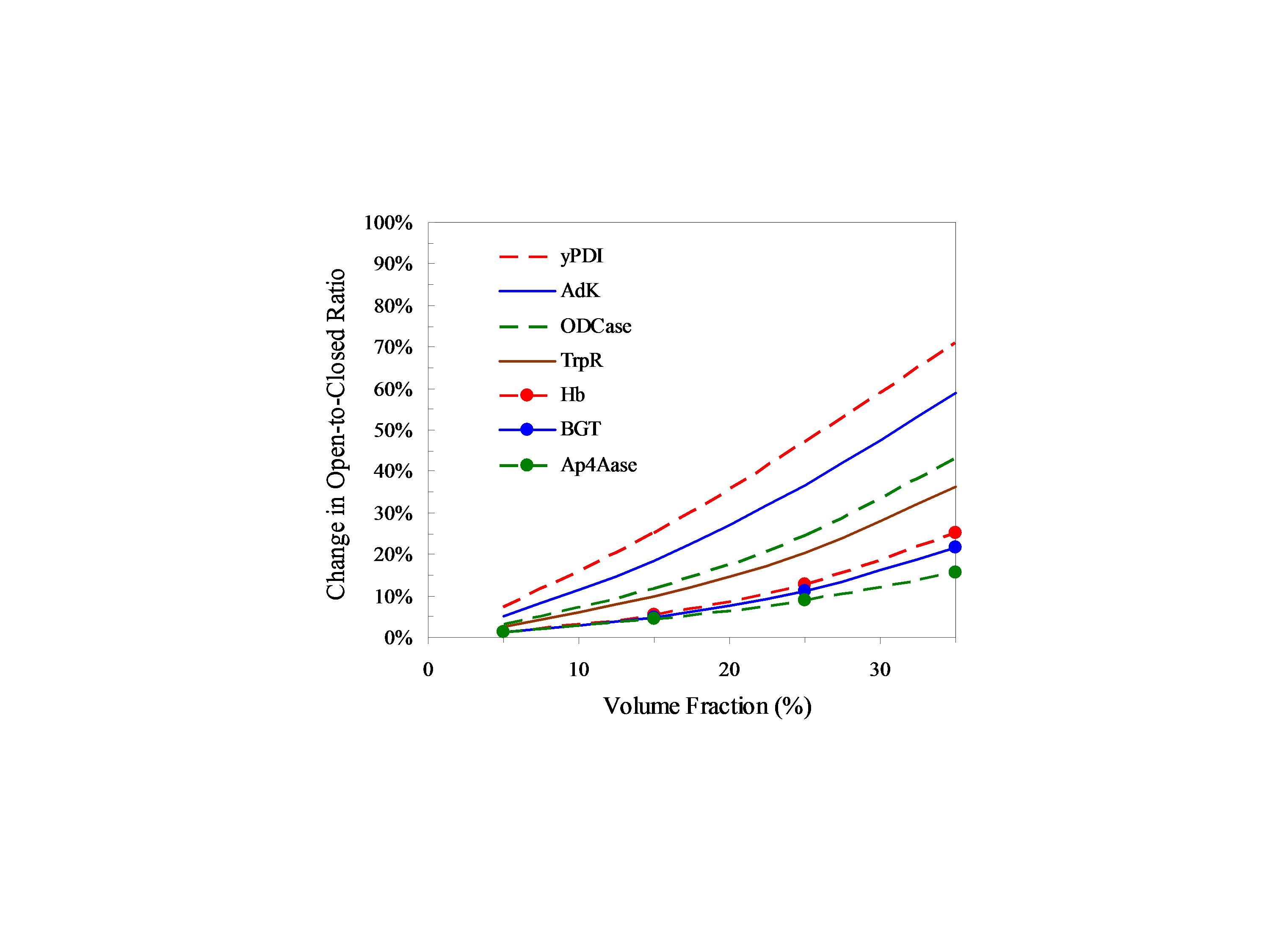

Supplement: Figure S2 — Effects of crowding on the open-to-closed population ratios of seven proteins. The crowder radius is 30 Å. (0.54 MB TIF) [file pcbi.1000833.s002.tif]

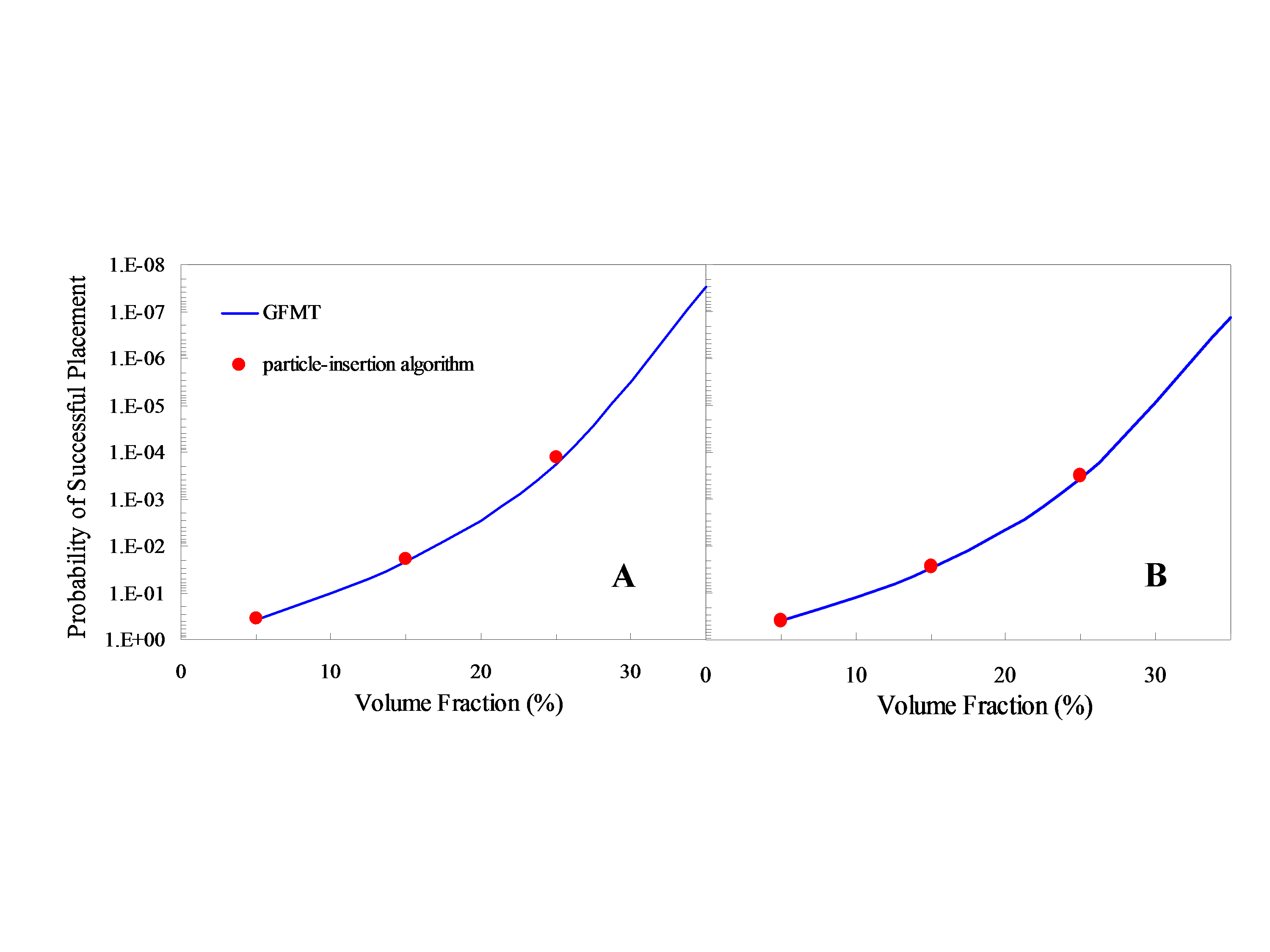

Supplement: Figure S3 — Comparison of GFMT predictions and results obtained by the particle-insertion algorithm. (A) Crowding-induced change, Δμo, in the chemical potential of AdK in the open state. (B) Corresponding quantity in the closed state. The crowder radius is 15 Å. (0.52 MB TIF) [file pcbi.1000833.s003.tif]

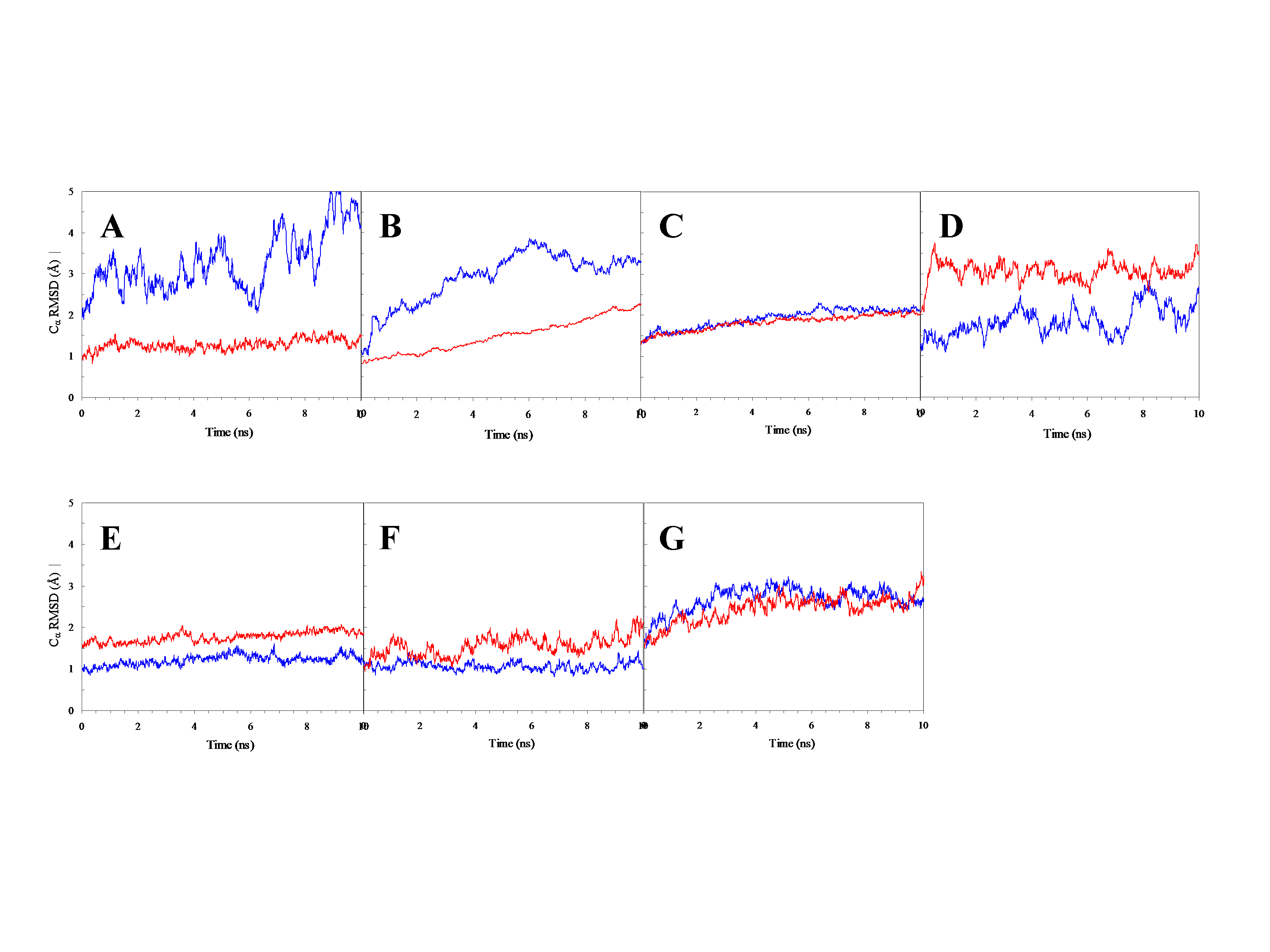

Supplement: Figure S4 — Root-mean-square-deviations of conformations during simulations from the starting X-ray or NMR structures. (A) AdK. (B) yPDI. (C) ODCase. (D) TrpR. (E) Hb. (F) BGT. (G) Ap4Aase. For each protein, Cα RMSDs of the open and closed states are displayed in blue and red, respectively. For ODCase, results averaged over four independent trajectories are shown. (0.55 MB TIF) [file pcbi.1000833.s004.tif]
