# Supplementary material for: Effects of Macromolecular Crowding on Protein Conformational Changes
Source: PLoS Comput Biol. 2010 Jul 1;6(7):e1000833. doi: 10.1371/journal.pcbi.1000833 (PMC2895631; doi:10.1371/journal.pcbi.1000833)
Supplement: Table S1 — Geometrical parameters of seven proteins for the prediction of Δμo and Δμc by the generalized fundamental measure theory (0.07 MB DOC) [file pcbi.1000833.s005.doc]

**Table S1.** Geometrical parameters of seven proteins for the prediction of Δ*μ*_o_ and Δ*μ*_c_ by the generalized fundamental measure theory

|  | *R*_c_ (Å) | Adk | yPDI | ODCase | TrpR | Hb | BGT | Ap_4_Aase |
| --- | --- | --- | --- | --- | --- | --- | --- | --- |
| *l*_p_ (Å) | 15 | 25.9  23.8 | 41.7  38.8 | 34.1  33.1 | 21.1  20.0 | 32.3  32.0 | 30.3  30.3 | 23.4  22.6 |
|  | 20 | 26.2  24.0 | 42.1  39.5 | 34.6  33.5 | 21.3  20.3 | 32.5  32.2 | 30.7  30.4 | 23.7  22.8 |
|  | 30 | 26.6  24.3 | 42.8  40.4 | 35.2  34.0 | 21.7  20.6 | 32.8  32.5 | 31.1  30.9 | 24.1  23.1 |
|  | 50 | 27.0  24.6 | 43.7  41.6 | 35.8  34.5 | 22.0  21.0 | 33.1  32.8 | 31.6  31.4 | 24.6  23.5 |
| *s*_p_ (Å^2^) | 15 | 7663.1  6817.3 | 17718.4  16502.4 | 13356.2  12845.5 | 4854.8  4449.5 | 13065.9  12871.6 | 10506.2  10316.8 | 6161.6  5996.8 |
|  | 20 | 7685.4  6836.5 | 17953.3  16413.5 | 13375.8  12850.6 | 4885.3  4475.4 | 13031.0  12846.7 | 10502.5  10320.4 | 6189.9  6022.5 |
|  | 30 | 7759.2  6891.1 | 18061.7  16459.3 | 13466.4  12915.4 | 4945.2  4526.2 | 13052.7  12875.8 | 10556.4  10367.2 | 6254.8  6078.8 |
|  | 50 | 7878.4  6974.1 | 18493.2  16666.1 | 13617.6  13054.1 | 5025.9  4594.6 | 13126.2  12956.8 | 10691.3  10475.4 | 6345.1  6153.3 |
| *v*_p_ (Å^3^) | 15 | 46841.8  43624.6 | 54127.1  51435.7 | 108223.4  105630.3 | 23871.5  21680.7 | 120261.5  117696.4 | 76938.6  74953.3 | 35543.9  35793.1 |
|  | 20 | 49113.7  45134.2 | 116037.1  117207.6 | 112672.1  109492.7 | 25119.4  22602.8 | 123888.0  121116.7 | 80142.9  78109.4 | 37111.5  37145.6 |
|  | 30 | 52172.7  47075.6 | 128023.3  127063.0 | 118994.5  114765.3 | 26698.5  23790.0 | 128156.9  125219.2 | 84653.9  82564.0 | 39093.2  38805.4 |
|  | 50 | 55639.6  49145.0 | 146214.4  139728.6 | 126377.9  120876.8 | 28358.0  25035.8 | 132342.2  129209.4 | 89337.5  87084.2 | 41191.3  40468.6 |

The crowder radius *R*_c_ serves as the probe radius in defining the crowder-exclusion surface; *s*_p_ is the area of this surface, *v*_p_ is the volume enclosed by this surface, and *l*_p_ is the radius of gyration calculated on points uniformly distributed on this surface. In each cell below a protein name, two entries show the averages of *l*_p_, *s*_p_, or *v*_p_ in the open and closed states. Averaging is over 100 representative conformations for all proteins but ODCase; for that protein the averaging is over 400 conformations from four independent trajectories. The variance for each entry is < 1.2% of the average.
